# Exemplifying interspecies variation of liposome *in vivo* fate by the effects of anti-PEG antibodies

**DOI:** 10.1016/j.apsb.2024.07.009

**Published:** 2024-08-05

**Authors:** Ercan Wu, Juan Guan, Yifei Yu, Shiqi Lin, Tianhao Ding, Yuxiu Chu, Feng Pan, Mengyuan Liu, Yang Yang, Zui Zhang, Jian Zhang, Changyou Zhan, Jun Qian

**Affiliations:** a School of Pharmacy, Key Laboratory of Smart Drug Delivery (Fudan University), Ministry of Education & Department of Pharmacy, Huashan Hospital, Fudan University, Shanghai 201203, China; b Department of Pharmacy, Shanghai Pudong Hospital, Pudong Medical Center & Department of Pharmacology, School of Basic Medical Sciences & State Key Laboratory of Molecular Engineering of Polymers, Fudan University, Shanghai 200032, China; c Department of Medical Oncology, Fudan University Shanghai Cancer Center, Shanghai 200032, China

**Keywords:** Lipid, Nanomedicine, Liposome, Interspecies, Anti-PEG antibody, Complement system, ABC phenomenon, *In vivo* fate

## Abstract

The different fate of liposomes among species has been discovered and mentioned in many studies, but the underlying mechanisms have not been explored. In the present work, we concentrated on the *in vivo* fate of PEGylated liposomes (sLip) in three commonly used species (mice, rats, and dogs). It was exhibited that the accelerated blood clearance (ABC) phenomenon and hypersensitivity in large animals (beagle dogs) were much more significant than that in rodents. We demonstrated that anti-PEG IgM (partially) and complement (mostly) determined the elimination of sLip and linked the distinct interspecies performances with the diverse complement capacity among species. Based on the data from animals and clinical patients, it was revealed that the fate of sLip in large animals was closer to that in humans, for the sufficient complement capacity could expose the potential adverse reactions caused by anti-PEG antibodies. Our results suggested that the distinctive interspecies performances of sLip were highly related to the physiological variabilities among species, which should not be overlooked in the innovation and translation of nanomedicines.

## Graphical abstract

**Figure:**
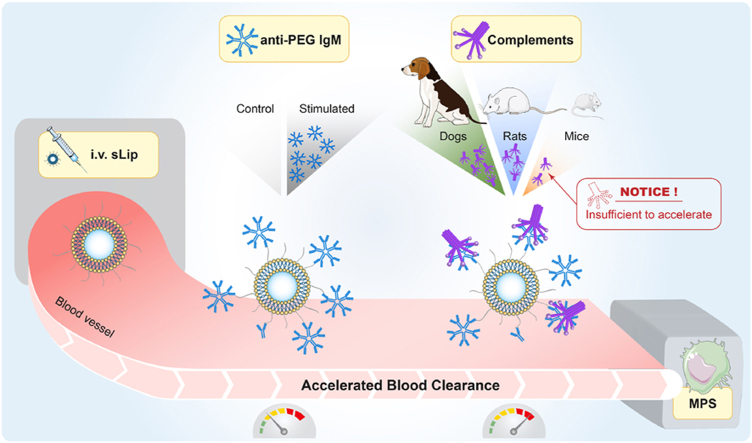

## 1 Introduction

Liposomes are one of the most mature nanocarriers in preclinical research and clinical applications. The special vesicle structure formed by the lipid bilayer endows liposomes with high versatility for encapsulating both hydrophilic and hydrophobic payloads, improving the pharmacokinetic and biosafety profiles^1^, ^2^, ^3^. However, clinical translation of liposomal formulations remains challenging, for only less than 20 types approved for marketing^4^^,^^5^. It is essential to narrow the gap in bench-to-bedside translation by understanding the regulation of liposomes by organisms in preclinical studies. In the process of clinical translation, it is crucial to explain the interspecies relevance of the *in vivo* performance of liposomes, which ensures the guiding value of preclinical studies for clinical applications^6^, ^7^, ^8^. Nevertheless, the *in vivo* fate of liposomes is much more complicated than that of ordinary formulations for unclear regulatory effects and interspecies physiopathological variabilities^9^, ^10^, ^11^, ^12^, ^13^. So far, the mechanistic understanding of interspecies variations of the *in vivo* fate of liposomes has not been elucidated clearly.

Among all liposomal therapeutics, PEGylation is prevalently used to prolong the circulation duration of liposomes in peripheral blood and to mitigate the uptake and rupture by the mononuclear macrophage system (MPS)-related cells^14^^,^^15^. Although PEGylated products were once thought to be non-immunogenic, many studies revealed that anti-PEG antibodies were produced to respond to the stimulation, which were observed in many animal species^16^, ^17^, ^18^. Moreover, it was reported that anti-PEG antibodies increasingly occurred in humans without receiving PEGylated agents, which was speculated to be related to the boosting exposure of cosmetics, drugs, and food with the addition of PEG and PEG derivatives^18^, ^19^, ^20^, ^21^. The remarkable thing is that anti-PEG antibodies would possibly result in the rapid elimination of PEG-modified therapeutics from the peripheral blood, which has been observed in many species and is referred to as the accelerated blood clearance (ABC) phenomenon^22^^,^^23^.

The possible mechanism of the ABC phenomenon is that anti-PEG antibodies recognize and bind to PEGylated therapeutics, rapidly activating complement to accelerate removal of PEGylated therapeutics from the peripheral blood^24^^,^^25^. This unanticipated phenomenon alters the pharmacokinetics (PK) and pharmacodynamics (PD) of PEGylated liposomes significantly. In addition, according to the clinical data, the influences of complement activation after recognition by anti-PEG antibodies include a shortened half-life period, enhanced liver accumulation, elevated risk of infusion reactions and skin toxicities, reduced drug efficacy and so on^17^^,^^21^. Therefore, the application of PEGylated therapeutics is facing new challenges and many studies have focused on the effect of anti-PEG antibodies and the relevant complement system on PEGylated therapeutics. A better understanding of interspecies differences paves an avenue to clarify the *in vivo* regulatory mechanisms and achieve interspecies conversion of the *in vivo* fate of liposomes, which is essential to bridge the gap in bench-to-bedside translation and fulfill the prediction of efficacy and adverse reactions.

In this present work, mice, rats, and beagle dogs were selected due to their widespread application in the preclinical evaluation of PEGylated liposomes^26^, ^27^, ^28^. In different species, the pharmacokinetics profiles of PEGylated liposomes (sLip) were investigated and compared with or without the presence of anti-PEG antibodies among all species. The interspecies differences in pharmacokinetics and adverse reactions were noticed and the potential regulating mechanism was illustrated. We also analyzed the relevance between preclinical animal models and clinical data and suggested possible solutions for achieving predictive efficacy and preventing adverse reactions.

## 2 Materials and methods

### 2.1 Reagents and antibodies

Hydrogenated soy phosphatidylcholine (HSPC, 92128-87-5), cholesterol (CHO, 57-88-5), and mPEG_2000_-DSPE (147867-65-0) were acquired from A.V.T. Pharmaceutical Co., Ltd. (Shanghai, China). Doxorubicin hydrochloride (MB1087), daunorubicin hydrochloride (MB1074) and DiD (DiIC18(5), 1,1′-dioctadecyl-3,3,3′,3′-tetramethylindodicarbocyanine perchlorate, MB6190) were acquired from Dalian Meilun Biotechnology Co., Ltd. (Dalian, China). Sephadex® G-50 (G5080) was purchased from Sigma–Aldrich (St. Louis, MO, USA). Goat anti-mouse IgM mu chain (HRP) (ab97230), goat anti-rat IgM mu chain (HRP) (ab97180), goat anti-dog IgM H&L (HRP) (ab112835), goat anti-human IgM mu chain (HRP) (ab97205), anti-C3 antibody (ab200999) were purchased from Abcam (Cambridge, MA, USA). 3,3′,5,5′-Tetramethylbenzidine (TMB) chromogen solution for ELISA assay (P0209), goat Anti-Rabbit IgG(H + L) (HRP) (A0208) and Fast Silver Stain Kit (P0017S) were purchased from Beyotime Biotechnology (Shanghai, China). Gradient precast polyacrylamide gels (4%–20%) (Cat# 456–1093) and protein dual color standards (Cat# 1610374) were acquired from BIO-RAD (Hercules, CA, USA). HisSep Ni-NTA agarose resin 6FF (20503ES10), ampicillin (60203ES10), and polymyxin B sulfate (60242ES03) were acquired from YEASEN Biotech (Shanghai, China). Duomeisu® was acquired from CSPC Pharmaceutical Group Ltd. (Shijiazhuang, China).

### 2.2 Animals and human serum

Adult male ICR and C57BL/6 mice, Sprague–Dawley rats were acquired from Shanghai SLAC Laboratory Animal Co., Ltd. (Shanghai, China) and kept under SPF condition. C3 knockout mice were acquired from Cyagen Biosciences Inc. (Suzhou, China) and kept under SPF conditions. Beagle dogs were acquired from Shanghai Jiaoda Nongsheng Experimental Practice Field Co., Ltd. (Shanghai, China) and kept under conventional conditions. All animal experiments were carried out by the Guidelines of the Care and Use of Laboratory Animals of Fudan University and approved by the Animal Ethics Committee of Fudan University.

The study protocol was reviewed and approved by the Ethics Committee of Fudan University Shanghai Cancer Center (NCT05354076). The trial was conducted according to the guidelines of Good clinical practice (GCP) and monitored by the GCP unit at the hospital. Participants provided written informed consent prior to taking part in the study.

### 2.3 Preparation and characterization of liposomes

sLip was prepared by the thin-film hydration and extrusion method. A mixture of HSPC/CHO/mPEG_2000_-DSPE (molar ratio of 57:38:5) dissolved in chloroform was rotary evaporated to form a thin film. Overnight after vacuuming, the film was hydrated with saline at 60 °C and successively extruded through polycarbonate membranes with pore diameters of 200, 100, and 50 nm also at 60 °C. The DiD-labelled sLip (sLip/DiD) was prepared by the same method except that DiD was added to the mixture before the formation of a thin film (0.4 mg DiD per 9.58 mg HSPC). After the extrusion, free DiD was removed by the Sephadex® G-50 column. The lipid concentration of liposomes was measured by phosphorus assay. The size, size distribution, and zeta potential of liposomes were detected in deionized water at a lipid concentration of 0.25 mmol/L by ZetasizerNano ZS90 (Malvern Instruments, Southborough, MA, USA).

### 2.4 Stimulation of anti-PEG antibodies

Mice (5 mg HSPC/kg), rats (2.5 mg HSPC/kg), and beagle dogs (1 mg HSPC/kg) were stimulated with plain sLip intravenously. The dosages of liposomes among species were calculated according to interspecies dose exchanging index^29^, which were one-tenth of their regular pharmacokinetic doses. After 5–7 days, blood was sampled and serum was collected after centrifugation at 3000 rpm for 8 min (Eppendorf, Centrifuge 5417R, Hamburg, Germany). Titers of anti-PEG IgM/IgG in the serum of mice, rats, and dogs were measured by ELISA assay using mPEG_2000_-DSPE as antigens.

Also, in mice and rats, different doses of sLip were injected to stimulate different levels of antibodies (0.5 and 50 mg HSPC/kg for mice, 0.25 and 25 mg HSPC/kg for rats).

### 2.5 ELISA assay

The anti-PEG IgM/IgG in serum was detected by ELISA, which is widely applied to quantify levels of antibodies due to its sensitivity and intuitiveness^17^. 96-well ELISA plate was coated with mPEG_2000_-DSPE in ethyl alcohol (2 μg/well). After drying overnight, all wells were rinsed with 0.1% Tween-20 in PBS (PBST) 3 times and blocked with 5% BSA (150 μL/well) at room temperature for 1 h to reduce unspecific absorption. The serum was gradually diluted with 0.1% BSA and incubated in the blocked wells at 37 °C for 1 h. After rinsing with PBST (200 μL/well) 3 times, a secondary HRP-conjugated antibody diluted with 0.1% BSA was added (100 μL/well) and incubated at 37 °C for 1 h. After rinsing, TMB solution was added to the wells for 8 min incubation, and the reaction was terminated by 0.18 mol/L H_2_SO_4_. UV absorbance at 450 nm of each well was measured and the titers of anti-PEG IgM/IgG were calculated by GraphPad Prism 8.0.1 software.

### 2.6 Pharmacokinetic studies

Mice, rats, and beagle dogs were divided into two groups randomly (*n* = 4–5). One group was stimulated by sLip to produce pre-existing anti-PEG antibodies and the other group was injected with saline in the same volume as a control group. After 5–7 days, all mice (50 mg HSPC/kg for regular dose and 5 mg HSPC/kg for low dose), rats (25 mg HSPC/kg for regular dose and 2.5 mg HSPC/kg for low dose), and beagle dogs (10 mg HSPC/kg) were intravenously injected with sLip/DiD. The dosages of liposomes among species were calculated according to interspecies dose exchanging index^29^. At predominated time points, blood was sampled and plasma was separated by centrifugation at 3000 rpm for 8 min (Eppendorf). The plasma concentration of sLip/DiD was detected by a fluorescence spectrophotometer (Ex/Em at 630 nm/675 nm). Pharmacokinetic parameters were calculated by GraphPad Prism 8.0.1 software and PK Solver 2.0^30^.

### 2.7 Expression and characterization of PEG-scFv

Anti-PEG single chain variable fragment antibody (PEG-scFv) was applied to sediment PEGylated liposomes by simple incubation and low-speed centrifugation, which was introduced in our previous work^31^^,^^32^. The PEG-scFv plasmid was constructed and cloned into competent *E. coli*. After culturing overnight on the plate, several individual clones were selected and amplified. The expressed *E. coli* were collected after centrifugation at 4 °C, 5000×*g* for 30 min (Thermo Fisher Scientific, Sorvall Biofuge Stratos, Waltham, MA, USA) and resuspended in lysis buffer (0.5 mol/L NaCl and 0.1 mol/L polymyxin B sulfate). Then the supernatant was obtained after centrifugation at 4 °C, 12,000×*g* for 10 min (Thermo Fisher Scientific). PEG-scFv was purified by passing through Ni-NTA agarose resin and eluted with elution buffer (250 mmol/L imidazole in PBS, pH 8.0). The eluted fractions were concentrated at 4000×*g* (Thermo Fisher Scientific) and washed with chilled PBS until the storage buffer had been completely replaced. A BCA protein assay kit was used to detect the concentration of PEG-scFv.

### 2.8 Separation and characterization of protein corona

In a 3 mL gravity chromatography column, Ni-NTA agarose resin was filled in and equilibrated with 5 mL PBS 5 times. Liposomes (20 μL) were incubated with PEG-scFv (60 μL, 0.2 mg/mL)^31^ at room temperature for 10 min, followed by the addition of 20 μL baseline serum of cancer patients to form protein corona with 30 min incubation at 37 °C. The mixture was loaded into the column gently and incubated with Ni-NTA resin at room temperature for 10 min. After the incubation, unbound liposomes and redundant proteins were thoroughly washed with 200 μL washing buffer (5 mmol/L imidazole in PBS, pH 8.0) 15 times. PEG_8000_ in PBS (10 mg/mL) was added to elute the liposomes with plasma protein and fractions were collected at a volume of 200 μL per EP tube. The protein concentration of each tube was determined by a BCA kit and fluorescence intensity was measured by a fluorescence spectrophotometer (Ex/Em at 480 nm/550 nm).

Each sample of plasma protein (40 μL) was mixed with SDS-PAGE loading buffer (5 × , with DTT, 10 μL) and boiled at 100 °C for 10 min. Denatured proteins were separated by a 4%–20% gradient polyacrylamide gel and transferred to the PVDF membrane. Nonspecific binding sites on PVDF membrane were blocked by incubating with 5% BSA at room temperature for 1 h. The target protein was marked by the following incubation with a specific antibody conjugated with HRP overnight at 4 °C. The signal was imaged (Clinx Co., Ltd., ChemiScope 6000, Shanghai, China) and the result was analyzed by Image J software.

### 2.9 Peripheral blood cell analyses

Blood (200 μL) of dogs at 2 h after PK injection was collected into EP tubes with anticoagulant EDTA. All samples were kept at 4 °C until the routine blood test in the Department of Laboratory Animal Science of Fudan University.

### 2.10 Complement activation assay

Sheep red blood cells (SRBC) were washed by PBS twice and sensitized with 2U Hemolysin (1:1, *v*/*v*) at 37 °C for 30 min. Then, the serum of each patient (70 μL) was gradually diluted and incubated with an equal volume of sensitized SRBC at 37 °C for 30 min. The same volume of PBS or deionized water was set as the negative and positive control to be mixed with SRBC, respectively. After the incubation, all samples were centrifuged at 1000×*g* for 5 min at 4 °C (Eppendorf), and the UV absorbance of the supernatant was detected at 542 nm. The hemolysis percentage was calculated as shown in Eq. (1):

(1) Hemolysis (%) = (*A*_sample_−*A*_PBS_)/(*A*_DW_−*A*_PBS_) × 100

### 2.11 Quantification of DOX in plasma

For DOX extraction from the plasma of patients, methanol with daunorubicin hydrochloride (10 μg/mL) as internal standard and chloroform (400 μL) were added into the plasma and mixed. The suspension was centrifuged (10,000×*g*, 4 °C) for 5 min (Eppendorf), and the supernatant was collected and dried, which was re-dissolved in acetonitrile (30% in ultrapure water). After vortex and centrifugation, the solution was analyzed by HPLC coupled with a fluorescent detector (Ex/Em at 480 nm/550 nm, C18 column, 40% acetonitrile elution).

LC‒MS/MS (Shimadzu, LCMS-8060, Kyoto, Japan) was used to detect doxorubicin concentration in blood samples when the concentration was lower than the limit of detection of HPLC. 0.1% formic acid in ultrapure water was used as mobile phase A and acetonitrile was used as mobile phase B. Gradient elution was performed as follows at a flow rate of 0.3 mL/min: 16% B for 2 min, 16%–34% B over 6 min, 34%–16% B over 0.1 min, and 16% B for 2.9 min. The transitions of *m*/*z* 544.30 → 397.25 for doxorubicin and *m*/*z* 528.30 → 321.10 for daunorubicin were used.

### 2.12 Statistical analysis

Data are presented as the means ± standard deviations (SD) and were analyzed by GraphPad Prism software 8.0.1 (GraphPad Software, San Diego, CA, USA). ∗*P*<0.05 was considered statistically significant (0.01<∗*P*<0.05, 0.001<∗∗*P*<0.01, 0.0001<∗∗∗*P*<0.001, ∗∗∗∗*P*<0.0001).

## 3 Results

### 3.1 Large animals demonstrate aggravated ABC phenomenon at the regular dose of sLip

**Figure 1:**
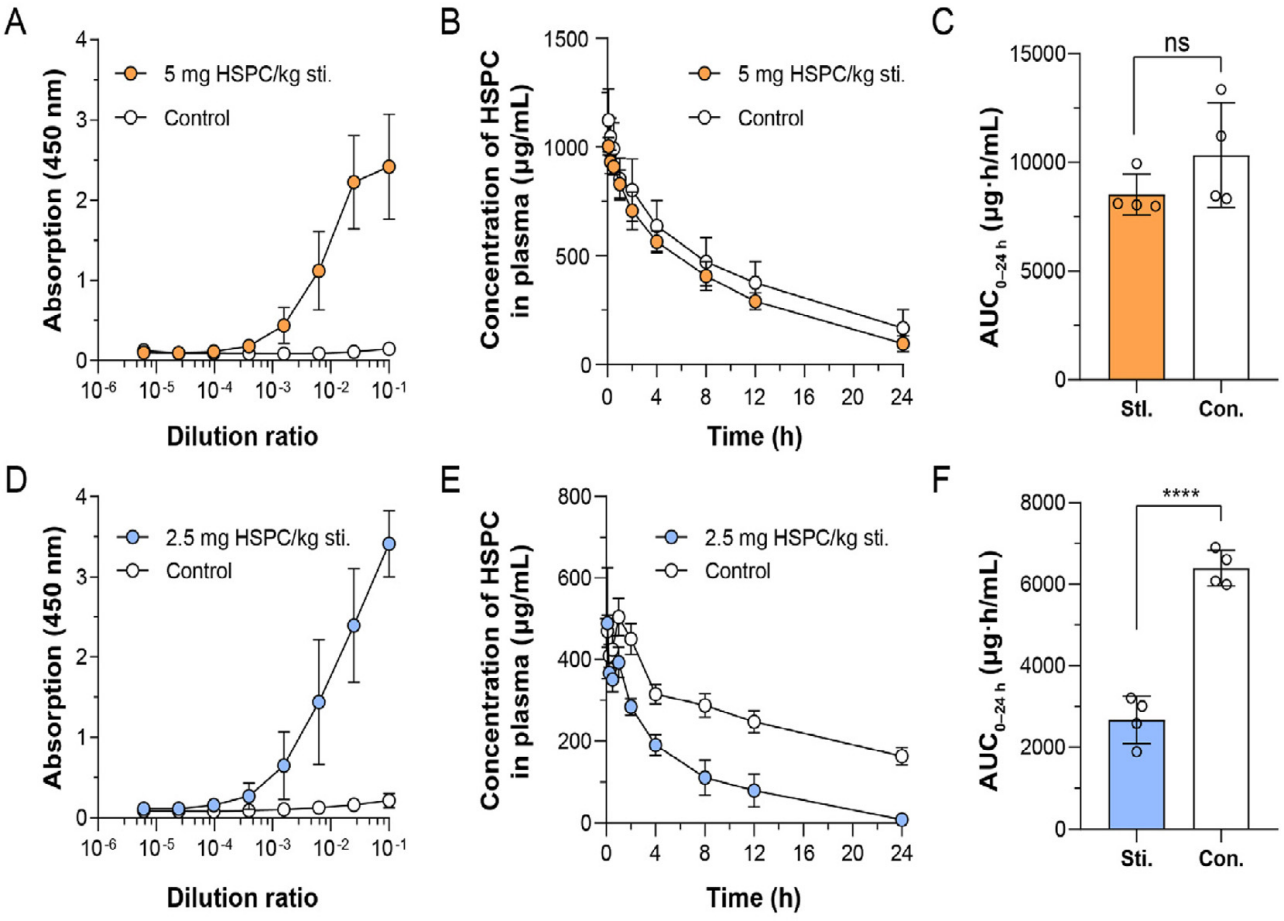
Interspecies difference of accelerated blood clearance (ABC) phenomenon among ICR mice (A–C) and SD rats (D–F). (A) Anti-PEG IgM was stimulated in mice after intravenous administration of sLip (5 mg HSPC/kg) and detected 5 days post-injection by ELISA (*n* = 13–16). (B) Plasma concentration of sLip at the regular dose (50 mg HSPC/kg) in the stimulated mice and the control mice was measured (*n* = 4). (C) The AUC_0–24 h_ of sLip at the regular dose was calculated in both stimulated mice and control mice, showing a 17.6% decrease (8519 *versus* 10336 μg·h/mL) caused by stimulation (*n* = 4). (D) Anti-PEG IgM was stimulated in rats after intravenous administration of sLip (2.5 mg HSPC/kg) and detected 5 days post-injection by ELISA (*n* = 8–10). (E) Plasma concentration of sLip at the regular dose (25 mg HSPC/kg) in the stimulated rats and the control rats was measured (*n* = 4). (F) The AUC_0–24 h_ of sLip at the regular dose was calculated in both stimulated rats and control rats, showing a 58.2% decrease (2673 *versus* 6393 μg·h/mL) caused by stimulation (*n* = 4). Data are mean ± SD and analyzed by GraphPad Prism 8.0. Statistical significance in the figure (C) and (F) was evaluated by *t*-test (ns, not significant. ∗∗∗∗*P* < 0.0001).

To study the interspecies difference of the ABC phenomenon, PEGylated liposomes (sLip) containing mPEG_2000_-DSPE (5% molar ratio) were prepared (see Methods) and characterized (see Supporting Information Table S1). Meanwhile, fluorescent dye DiD (0.4 mg DiD per 9.58 mg HSPC) labeled sLip was prepared using a similar method, in which DiD was readily anchored in the lipid bilayer with high stability^33^. In the pharmacokinetic study, the fluorescent intensity of DiD could be sensitively detected in plasma. On the other hand, ICR mice and SD rats were selected as representatives of different species for their widespread application in preclinical evaluation of sLip-based therapeutics, which were stimulated *via* intravenous injection of blank sLip (without payloads) at a dose of 5.0 mg lipid/kg of mice and 2.5 mg lipid/kg of rats (calculated according to interspecies dose exchanging index^29^), respectively. Blood was sampled 5–7 days after the injection and the titers of anti-PEG IgM in serum were measured using the ELISA method (see Methods). As shown in Fig. 1A and D, intravenous stimulation with blank sLip successfully induced significant production of anti-PEG IgM, registering a titer of 1:655 in mice and 1:2205 in rats. To study the interspecies difference of ABC phenomenon, both naïve (without stimulation of blank sLip and anti-PEG antibodies were negative) and pre-stimulated animals were intravenously injected with sLip/DiD at a regular dose (50 mg lipid/kg for mice and 25 mg lipid/kg for rats), which was converted as the equivalent dose in the clinical practice^29^^,^^34^. Blood was sampled at predetermined time points and plasma contents of sLip/DiD were measured by detecting the fluorescence intensity of DiD using a fluorescence spectrophotometer (see Methods). As shown in Fig. 1B and C, sLip was eliminated slightly faster (without significance) in the presence of the anti-PEG IgM, leading to a 17.6% decrease of the area under plasma concentration–time curve (AUC_0–24 h_, 8519 *versus* 10336 μg·h/mL) in comparison to that in the naïve mice.

However, the ABC phenomenon of sLip in rats was considerably more aggravated than that in mice (Fig. 1E and F). The AUC_0–24 h_ exhibited a 58.2% decrease in pre-stimulated rats (2673 *versus* 6393 μg·h/mL, *P*< 0.0001), which displayed a significant gap in the pharmacokinetic profiles of the two groups.

### 3.2 Mice eliminate sLip at the regular dose in an anti-PEG antibody-nondependent manner

**Figure 2:**
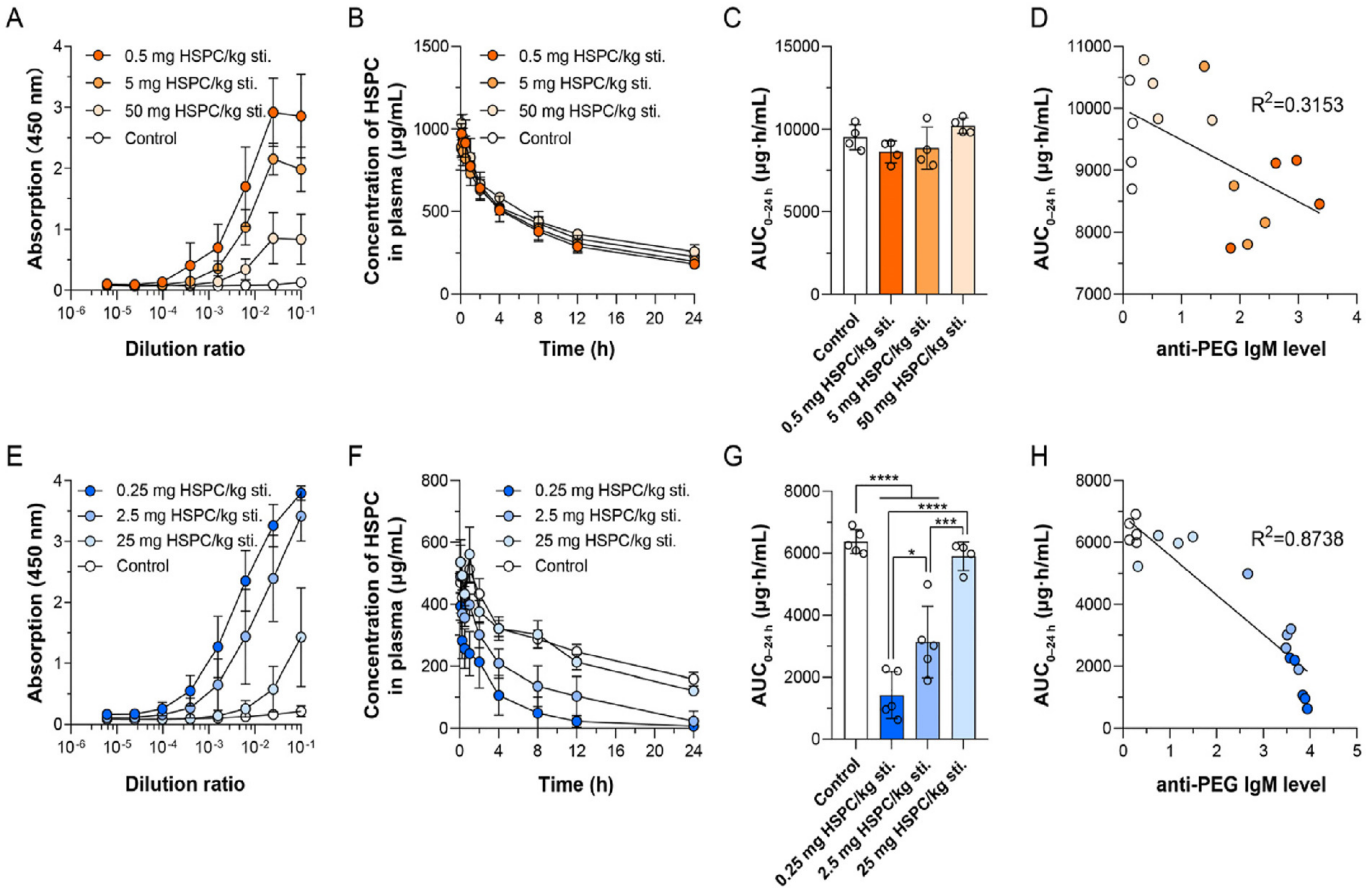
Effect of anti-PEG IgM titer on accelerated blood clearance (ABC) phenomenon of PEGylated liposomes (sLip) (at the regular dose) in ICR mice (A–D) and SD rats (E–H). (A) Different titers of anti-PEG IgM were stimulated by different doses of sLip in mice and were detected by ELISA (*n* = 8). (B) Plasma concentration of liposomes at the regular dose (50 mg HSPC/kg) in the stimulated mice (pre-stimulated with 0.5, 5 or 50 mg HSPC/kg sLip) and control group (*n* = 4). (C) The AUC_0–24__h_ of sLip (50 mg HSPC/kg) in mice with different levels of anti-PEG IgM was calculated and compared between groups (*n* = 4). (D) The correlation between the AUC_0–24__h_ of liposomes and the anti-PEG IgM level in mice was calculated. (E) Different titers of anti-PEG IgM were stimulated by different doses of sLip in rats and were detected by ELISA (*n* = 8–10). (F) Plasma concentration of liposomes at the regular dose (25 mg HSPC/kg) in the stimulated rats (pre-stimulated with 0.25, 2.5 or 25 mg HSPC/kg sLip) and control group (*n* = 4–5). (G) The AUC_0–24__h_ of sLip (25 mg HSPC/kg) in rats with different levels of anti-PEG IgM was calculated and compared between groups (*n* = 4–5). (H) The correlation between the AUC_0–24__h_ of liposomes and the anti-PEG IgM level in rats was calculated. Data were mean ± SD and analyzed by GraphPad Prism 8.0. Statistical significance in the figure (C) and (G) was evaluated by *t*-test (0.0001<∗∗∗*P*<0.001, ∗∗∗∗*P*<0.0001).

To investigate the detailed effect of anti-PEG IgM on the clearance of sLip, different anti-PEG IgM titers were stimulated in mice by intravenously injecting different doses of sLip (0.5, 5 and 50 mg lipid/kg of mice body weight). Blood was sampled at 5–7 days after stimulation and the titers were measured by ELISA. As shown in Fig. 2A, the titer of anti-PEG IgM in serum was about 1:2282, 1:355, and 1:206 which were stimulated by sLip at the dose of 0.5, 5, and 50 mg lipid/kg, respectively. It was consistent with previous reports that the higher level of anti-PEG IgM was stimulated by the lower dose of sLip in mice^23^^,^^35^. To study the influence of anti-PEG IgM titer on the ABC phenomenon, sLip/DiD was intravenously injected at a regular dose (50 mg lipid/kg) to both naïve mice and stimulated mice with different anti-PEG IgM titers. As shown in Fig. 2B and C, it was unexpected that the clearance rate of sLip/DiD barely varied regardless of anti-PEG IgM titers. The irrelevance between the AUC_0–24 h_ values and the anti-PEG IgM levels (the highest absorption of each sample was used, for the titers of the control group could not be calculated) suggested that sLip at the regular dose was eliminated in an anti-PEG IgM-nondependent manner in mice (Fig. 2D).

Meanwhile, to explore the impact of anti-PEG IgM in rats and compare the interspecies disparity of the ABC phenomenon between mice and rats, different doses of sLip were intravenously injected into rats to stimulate various anti-PEG IgM titers. As shown in Fig. 2E, the anti-PEG IgM titers in rat serum were 1:8742, 1:1772, and 1:57 which were respectively stimulated by sLip at the dose of 0.25, 2.5, and 25 mg lipid/kg. sLip/DiD at the regular dose (25 mg lipid/kg) was injected intravenously into both naïve rats and stimulated rats with various anti-PEG IgM titers. Blood was sampled at predetermined time points and the plasma concentration of sLip was measured by detecting the fluorescence intensity of DiD (see Methods). As shown in Fig. 2F and G, unlike the irrelevance between sLip clearance rate and IgM levels in mice, sLip elimination rate demonstrated a high correlation with anti-PEG IgM levels in rats (Fig. 2H).

According to the present study, after recognition of sLip by anti-PEG IgM, the classical pathway of the complement system was strongly activated, playing crucial roles in eliminating sLip *via* complement receptor-mediated pathway^23^. It was verified that the complement levels of mice were lower than other mammals, such as rats, rabbits, and humans^36^. Distinctive from mice, the rat complement was sufficient to be activated to accelerate sLip elimination in the presence of anti-PEG IgM. Therefore, the higher level of anti-PEG IgM in rats was able to cause more complement activation after binding to sLip and to exhibit a more apparent ABC phenomenon in rats. In contrast, anti-PEG IgM did not exhibit relevance with the sLip elimination in mice, which might be attributed to the limited complement capacity.

### 3.3 Complement determines *in vivo* fate of sLip in mice

**Figure 3:**
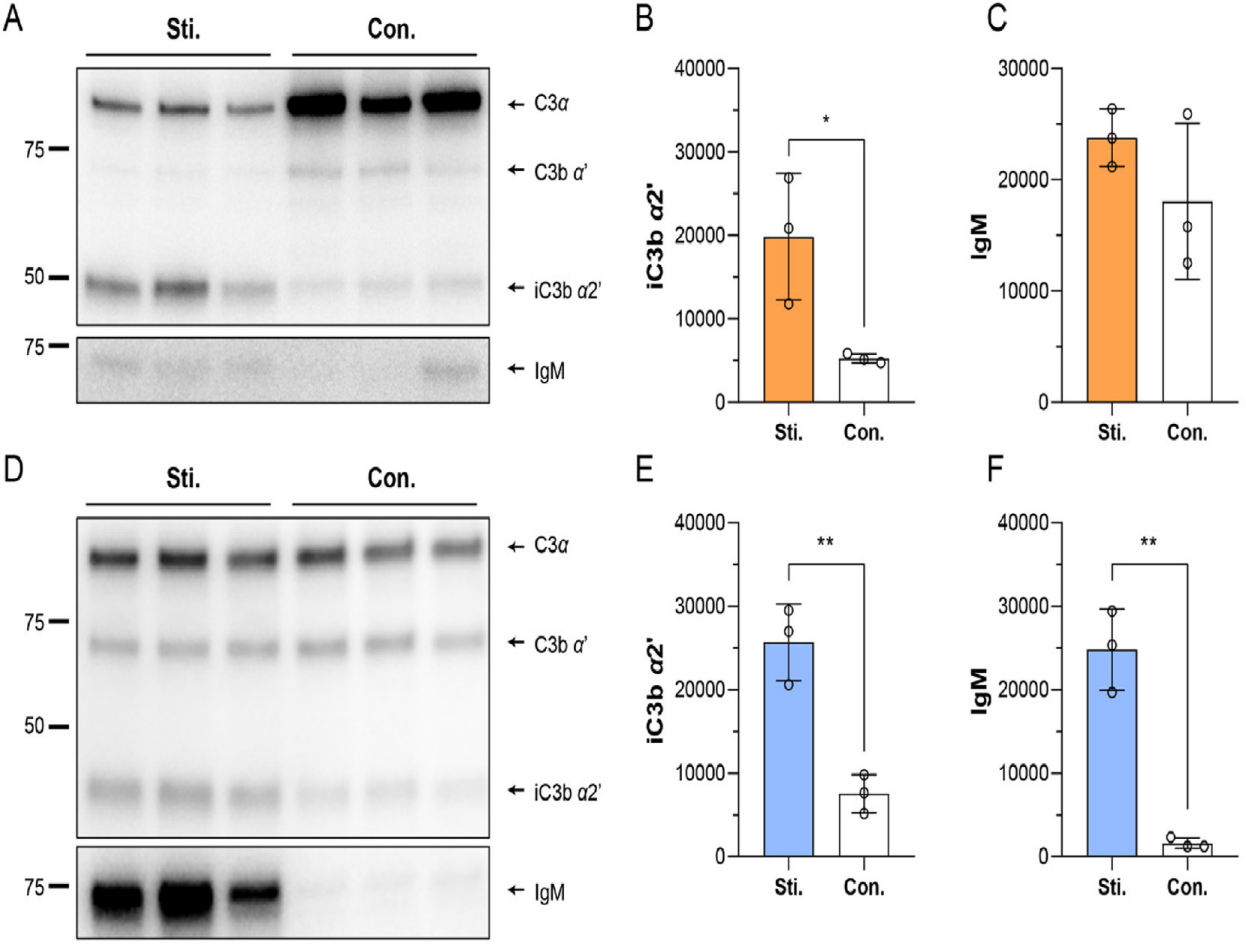
The protein corona of sLip formed in the stimulated and naïve serum of ICR mice (A–C) and SD rats (D–F). (A) Western blot analyses of C3*α*, C3b *α*', iC3b *α*2' and IgM in the corona formed on the liposomal surface after incubation with stimulated (Sti.) and naïve (Con.) mice serum for 1 h at 37 °C (*n* = 3). (B) The quantitation of iC3b *α*2' in the protein corona of sLip formed in mice serum on Western blot bands analyzed by Image J software (*n* = 3). (C) The quantitation of IgM in the protein corona of sLip formed in mice serum (*n* = 3). (D) Western blot analyses of C3*α*, C3b *α*', iC3b *α*2' and IgM in the corona formed on the liposomal surface incubated with stimulated and naïve rat serum for 1 h at 37 °C (*n* = 3). (E) The quantitation of iC3b *α*2' in the protein corona of sLip formed in rat serum (*n* = 3). (F) The quantitation of IgM in the protein corona of sLip formed in rat serum (*n* = 3). Data were mean ± SD and analyzed by GraphPad Prism 8.0. Statistical significance in the figure (B, C) and (E, F) was evaluated by *t*-test (0.01<∗*P*<0.05, 0.001<∗∗*P*<0.01).

To reveal the regulation of anti-PEG IgM and complement on the *in vivo* fate of liposomes, the plasma proteins deposited on the surface of sLip (so-called protein corona) were analyzed. As displayed in Fig. 3, the protein coronas on sLip formed in the naïve and stimulated serum of mice or rats were separated and characterized. The grayscale value of IgM by semiquantitative image analysis indicated that the produced anti-PEG IgM efficiently adsorbed on the liposomal surface both in mice (Fig. 3C) and rats (Fig. 3F). Meanwhile, the presence of anti-PEG IgM remarkably aggravated the cleavage of C3 after incubation with sLip, resulting in significant enhancement of the byproduct iC3b *α*2’ (Fig. 3B and E).

**Figure 4:**
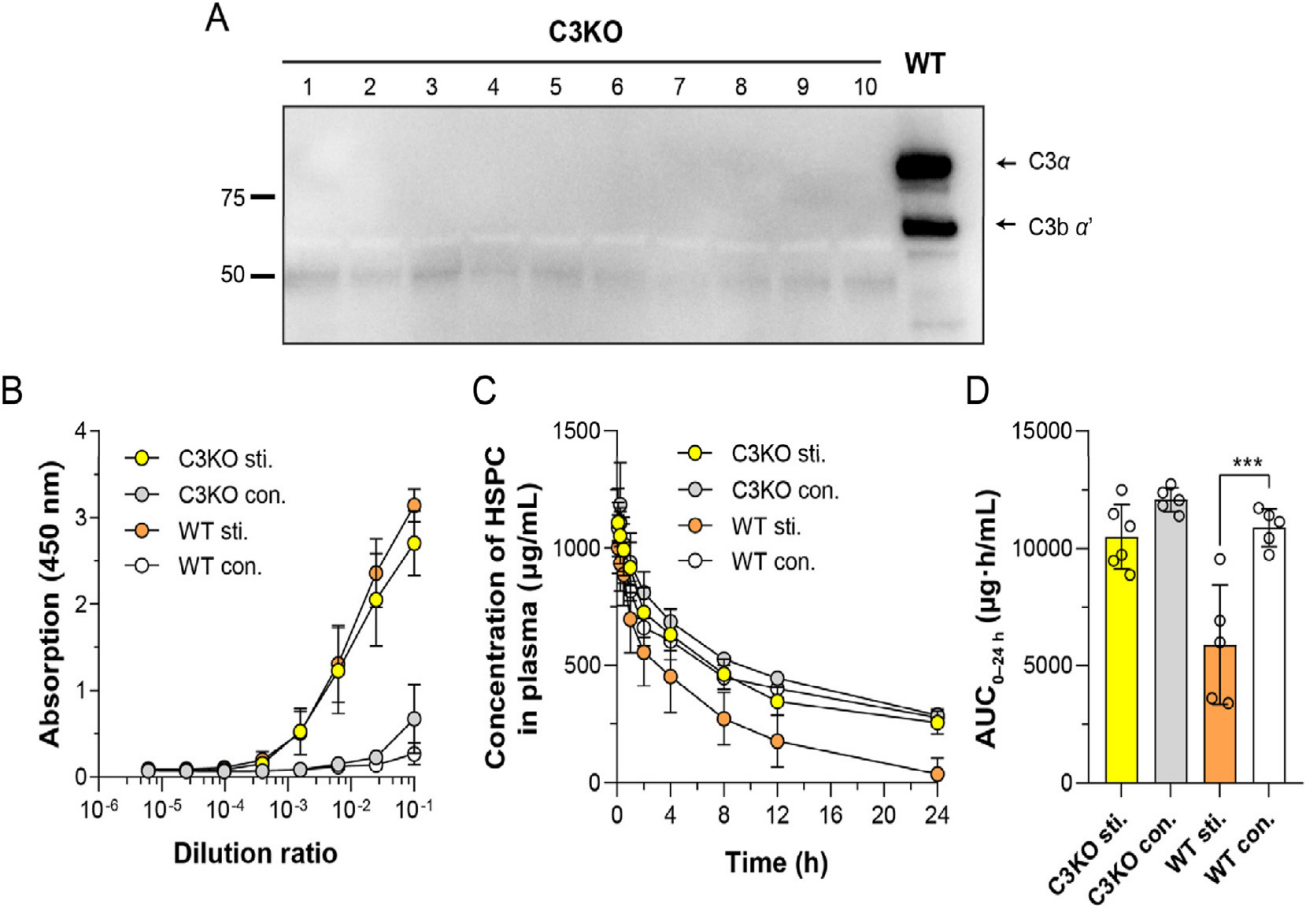
Effect of the complement 3 (C3) on blood clearance of sLip. (A) The deficiency of C3 in the plasma of C3 knockout (C3KO) mice was verified by Western blot (*n* = 10). (B) Anti-PEG IgM was stimulated by sLip (5 mg HSPC/kg) in C3KO and wide-type C57BL/6 mice (WT) and detected 5 days post-injection by ELISA (*n* = 5–9). (C) Plasma concentration of sLip at the regular dose (50 mg HSPC/kg) in the stimulated mice and control mice (*n* = 4–6). (D) The AUC_0–24__h_ of sLip was calculated and compared between groups. At the regular dose, stimulation with sLip resulted in a 13.1% decrease in AUC_0–24__h_ of C3KO mice (10499 *versus* 12078 μg·h/mL) and a 43.7% decrease in that of wide-type mice (6052 *versus* 10751 μg·h/mL) compared to their respective control groups (*n* = 4–6). Data are mean ± SD and analyzed by GraphPad Prism 8.0. Statistical significance in the figure (D) was evaluated by i-test (0.0001<∗∗∗*P*<0.001).

As aforementioned, C3 is the central and one of the indispensable participants in complement activation *via* all pathways^37^. Therefore, C3 knockout (C3KO, C3 expression was verified and shown in Fig. 4A) mice were used to verify the importance of the complement system in the ABC phenomenon of sLip. To exclude the interference of mouse strains, wild-type (WT) C57BL/6 mice were used as the control group. As shown in Fig. 4B, there was no impact on the production of anti-PEG IgM in C3KO mice in comparison to the wild-type mice after stimulation with blank sLip. In the pharmacokinetic study, sLip at the regular dose (50 mg lipid/kg) was intravenously injected into C3KO mice (naïve and stimulated) and WT mice (naïve and stimulated), respectively. As shown in Fig. 4C and D, the ABC phenomenon was attenuated in C3KO mice. The AUC_0–24 h_ decreased by only 13.1% in the stimulated C3KO mice, which was 43.7% in the naïve C57BL/6 mice (without pre-stimulation with blank sLip). It was worth noting that the ABC phenomenon in the naïve C57BL/6 mice was more significant than that in the naïve ICR mice (Fig. 1), which was attributed to the inter-strain difference and consistent with our previous reports^38^.

### 3.4 Complement capacity is the bottleneck for accelerated clearance of sLip in mice

**Figure 5:**
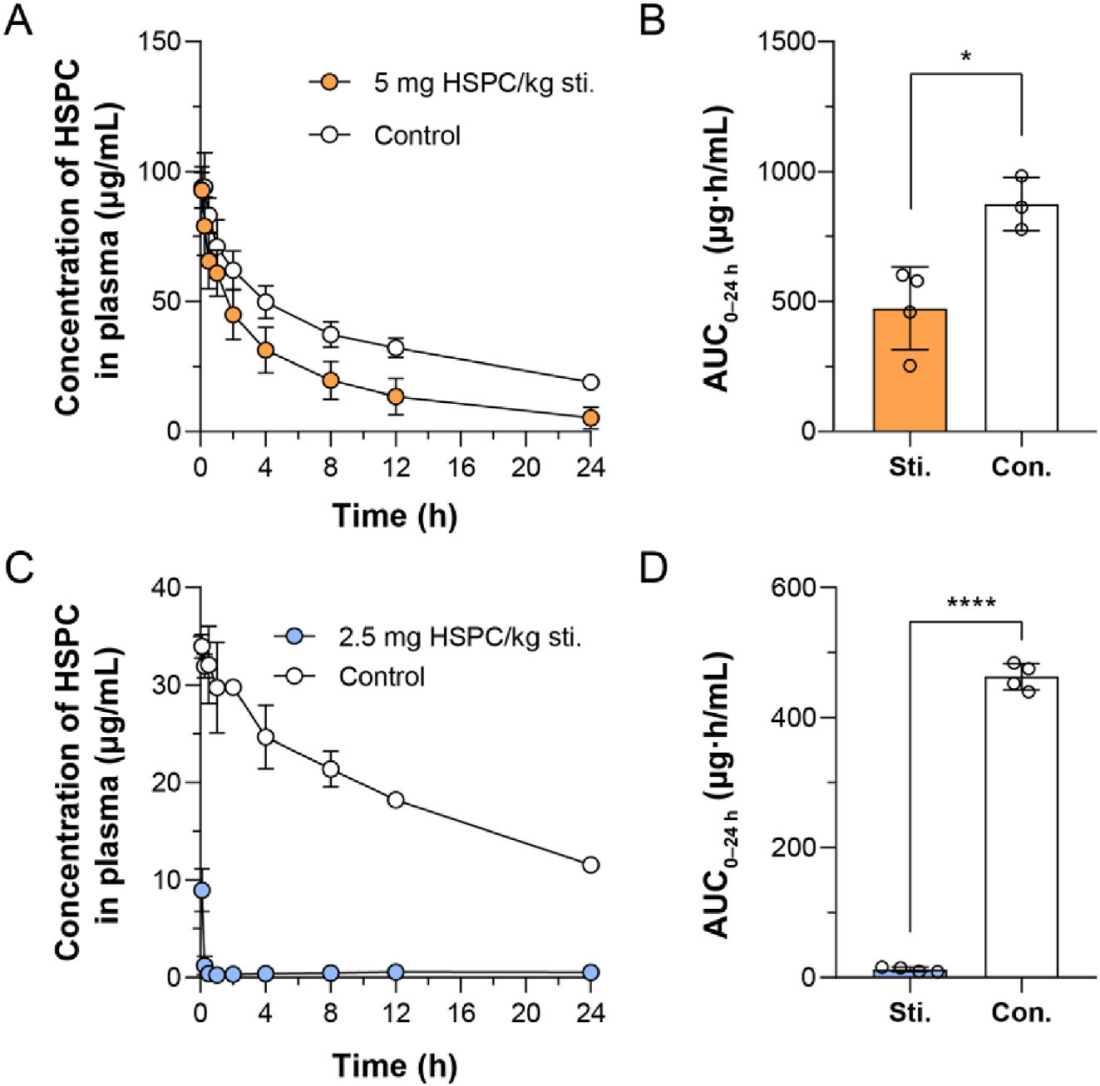
Effect of PEGylated liposomes (sLip) dose on accelerated blood clearance (A–C) phenomenon in ICR mice (A–B) and SD rats (C, D). (A) Plasma concentration of sLip at the low dose (5 mg HSPC/kg) in the stimulated mice and the control mice was measured (*n* = 3–4). (B) The AUC_0–24 h_ of sLip at the low dose was calculated in both stimulated mice and control mice, showing a 45.9% decrease (473.2 *versus* 874.7 μg·h/mL) caused by stimulation (*n* = 3–4). (C) Plasma concentration of sLip at the low dose (2.5 mg HSPC/kg) in the stimulated rats and the control rats was measured (*n* = 4). (D) The AUC_0–24 h_ of sLip at the low dose was calculated in both stimulated rats and control rats, showing a 97.4% decrease (12.12 *versus* 463.1 μg·h/mL) caused by stimulation (*n* = 4). Data are mean ± SD and analyzed by GraphPad Prism 8.0. Statistical significance in the figure (B) and (D) was evaluated by *t*-test (0.01<∗*P*<0.05, ∗∗∗∗*P*<0.0001).

To determine whether there was an insufficient presence of antibodies or complement, in other words, if the sLip were burdened with excessive particles impeding efficient clearance, sLip at the low dose (5 mg lipid/kg) was injected into both the stimulated and naïve mice to collect pharmacokinetic profiles (Fig. 5A and B). Although the level of anti-PEG IgM was equivalent, the blood clearance of low-dose sLip was more rapid (compared with the regular dose shown in Fig. 1B) and the ABC phenomenon was more obvious (45.9% decrease of AUC_0–24 h_). In C3KO mice, the results (Supporting Information Fig. S2) showed that sLip at the low dose can be removed by anti-PEG IgM solely to some extent, which implied that sLip in the blood can be synergistically eliminated by anti-PEG IgM (partially) and complements (dominantly). Also, sLip at the low dose (one-tenth of the regular dose, 2.5 mg lipid/kg) was injected into two groups of rats (the naïve and stimulated) for pharmacokinetic study (Fig. 5C and D). sLip at the low dose was eliminated from the blood circulation much more rapidly than that at the regular dose in rats in the presence of anti-PEG IgM (97.4% decrease of AUC_0–24 h_). The aggravated ABC phenomenon of sLip at the low dose (especially in mice) suggested that a high dose of liposomes may encounter insufficient complement capacity (when the anti-PEG IgM titers were equal and sufficient).

**Figure 6:**
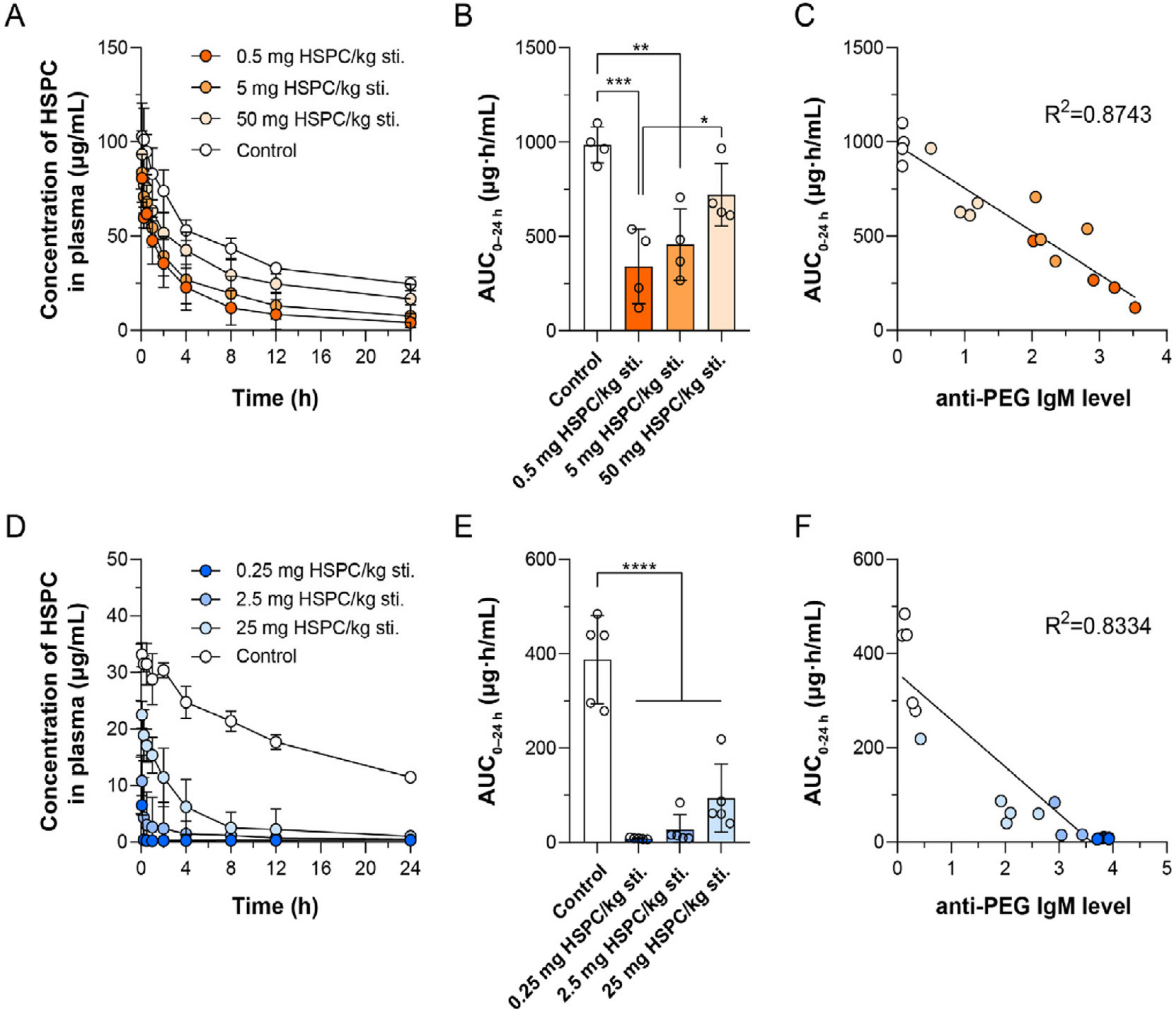
Effect of IgM titers on the accelerated blood clearance (ABC) phenomenon of PEGylated liposomes (sLip) at the low dose in ICR mice (A–C) and SD rats (D–F). (A) Plasma concentration of liposomes at the low dose (5 mg HSPC/kg) in the stimulated mice (pre-stimulated with 0.5, 5 or 50 mg/kg sLip) and control group (*n* = 4). (B) The AUC_0–24__h_ of sLip (5 mg HSPC/kg) in mice with different levels of anti-PEG IgM was calculated and compared between groups (*n* = 4). (C) The correlation between the AUC_0–24__h_ of liposomes and the anti-PEG IgM level in mice was calculated. (D) Plasma concentration of liposomes at the low dose (2.5 mg HSPC/kg) in the stimulated rats (pre-stimulated with 0.25, 2.5 or 25 mg/kg sLip) and control group (*n* = 4–5). (E) The AUC_0–24__h_ of sLip (2.5 mg HSPC/kg) in rats with different levels of anti-PEG IgM was calculated and compared between groups (*n* = 4–5). (F) The correlation between the AUC_0–24__h_ of liposomes and the anti-PEG IgM level in rats was calculated. Data were mean ± SD and analyzed by GraphPad Prism 8.0. Statistical significance in the figure (B) and (E) was evaluated by *t*-test (0.01<∗*P*<0.05, 0.001<∗∗*P*<0.01, 0.0001<∗∗∗*P*<0.001, ∗∗∗∗*P*<0.0001).

To gain further insights into the crucial impact of the downstream complement system on the elimination of sLip, low-dose sLip was injected into mice (5 mg lipid/kg) and rats (2.5 mg lipid/kg) with different titers of anti-PEG IgM, respectively. In sharp contrast with the regular dose (see Fig. 2B‒D), the clearance rate of sLip at the low dose was strongly correlated with the level of anti-PEG IgM in mice (Fig. 6A‒C). The higher titer of anti-PEG IgM induced enhanced sLip elimination because the complement capacity of mice was sufficient for the low dose of sLip. Therefore, the complement capacity in the IgM-complement axis was the bottleneck for accelerated blood clearance of sLip in mice. Meanwhile, the elimination of sLip at the low dose in rats was also highly correlated with the level of anti-PEG IgM (Fig. 6D‒F), in accordance with the result at the regular dose.

### 3.5 Beagle dogs demonstrate dramatic amplification of the ABC phenomenon and serve hypersensitivity reactions

Large animal models, such as canines, miniature swine, nonhuman primates, and so on, are always required to evaluate the pharmacokinetic and toxicity profiles in the preclinical evaluations^39^^,^^40^. Each species is anatomically, physiologically, and metabolically similar to humans at varying degrees. Due to more similar physical characteristics to humans and relatively cost-effective, canines remain the most popular nonrodent model of choice for the pharmacokinetic (and toxicity) assessments of most nanomaterial types^28^^,^^41^, ^42^, ^43^.

**Figure 7:**
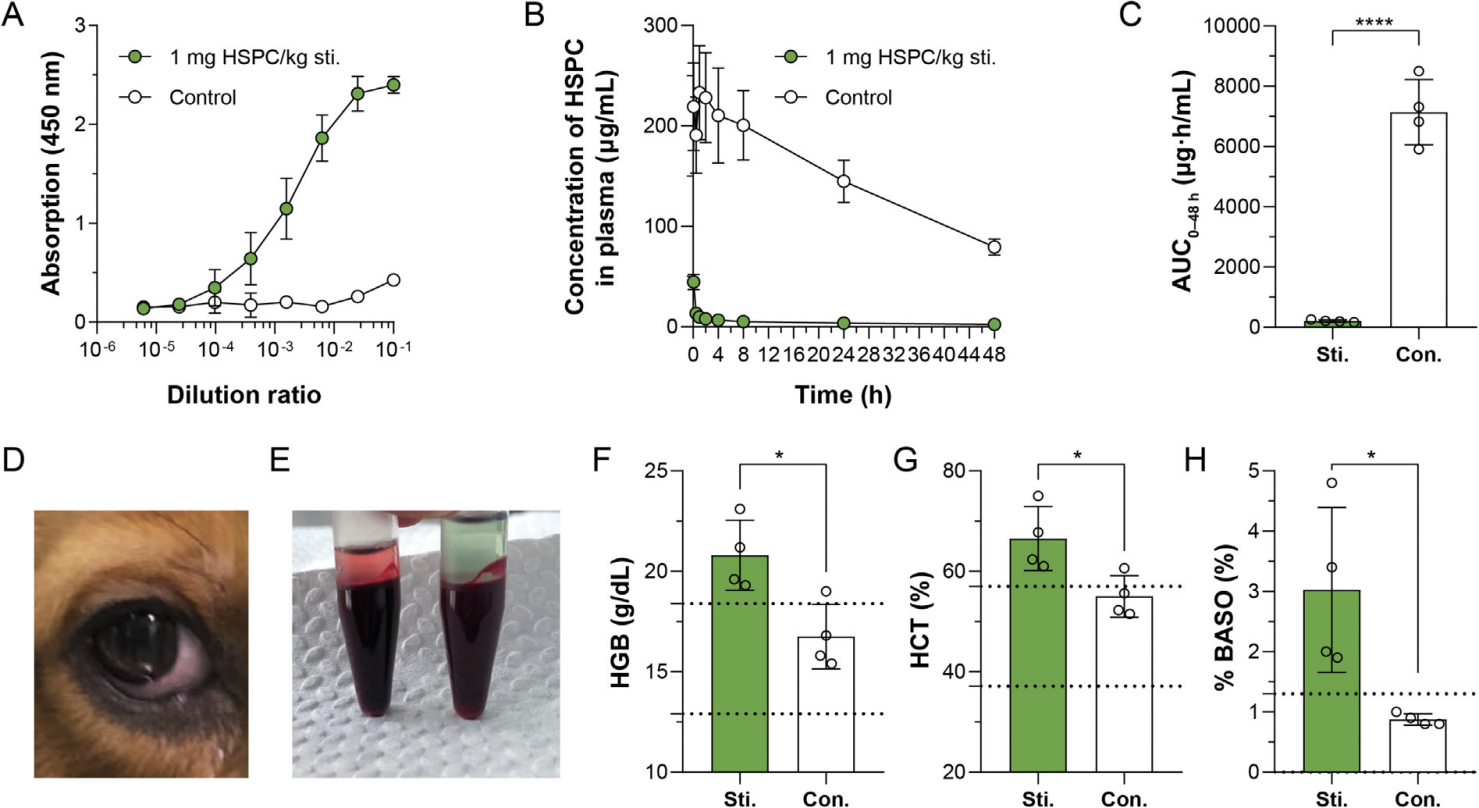
Effect of anti-PEG IgM on the *in vivo* performance of sLip in beagle dogs. Pharmacokinetics profiles (A–C) and adverse reactions caused by anti-PEG IgM (D–J) were recorded. (A) Anti-PEG IgM was stimulated in dogs after intravenous administration of sLip (1 mg/kg) and detected 5 days post-injection by ELISA (*n* = 4). (B) Plasma concentration of sLip at the regular dose (10 mg HSPC/kg) in the stimulated dogs and the control dogs was measured (*n* = 4). (C) The AUC_0–24 h_ of sLip at the regular dose was calculated in both stimulated dogs and control dogs, showing a 97.0% decrease (210.6 *versus* 7138 μg·h/mL) caused by stimulation (*n* = 4). A variety of symptoms such as (D) lethargy, (E) conjunctival congestion, (F) vomiting and (G) hemolysis arose in dogs with anti-PEG IgM after the injection of sLip at the regular dose (10 mg HSPC/kg). The peripheral blood cell analyses of the blood samples collected at 2 h after the injection of sLip displayed that the (H) hemoglobin content (HGB), (I) hematocrit value (HCT) and (J) the proportion of basophils (BASO%) in the stimulated dogs were abnormally increased (*n* = 4). Data are mean ± SD and analyzed by GraphPad Prism 8.0. Statistical significance in the figure (C) and (H–J) was evaluated by *t*-test (0.01<∗*P* < 0.05, ∗∗∗∗*P* < 0.0001).

To compare canines with rodents, beagle dogs were selected in the present work to investigate the pharmacokinetic profile and ABC phenomenon of sLip. Blank sLip at a dose of 1 mg lipid/kg (calculated according to interspecies dose exchanging index) was injected intravenously and blood was sampled at 5–7 days to measure the anti-PEG IgM titers in serum^29^. As shown in Fig. 7A, the stimulation successfully produced the anti-PEG IgM, registering a titer of 1:8245. In the following pharmacokinetic study, both naïve (without anti-PEG antibodies) and stimulated dogs were intravenously injected with sLip/DiD at a regular dose (10 mg lipid/kg) as the equivalent dose of 30 mg/m^2^ PLD in humans. Blood was sampled at predetermined time points and the plasma contents of sLip/DiD were measured. As shown in Fig. 7B and C, sLip was eliminated from the peripheral blood at an extremely rapid rate in the presence of anti-PEG IgM in beagle dogs. The AUC_0–48 h_ of sLip in the stimulated group was only 3.0% of that in naïve dogs. A huge disparity of plasma sLip concentration between the two groups existed from the very beginning of the PK study (5 min after administration, Fig. 7B). In contrast with the result in mice and rats (Fig. 1 and Supporting Information Table S3), the ABC phenomenon was dramatically augmented in beagle dogs, which displayed a tremendous gap in the pharmacokinetic profiles of the two groups even at the conventional equivalent dose. The protein coronas formed in the naïve and stimulated serum of dogs were also separated and characterized. As in rodents, more IgM was adsorbed on the surface of sLip in the stimulated serum, which caused stronger complement activation (Supporting Information Fig. S3).

Besides the dramatic amplification of the ABC phenomenon, several adverse reactions caused by anti-PEG IgM were found acutely in canines but not rodents. Unexpectedly, a variety of symptoms such as lethargy, vomiting, diarrhea, shortness of breath, and conjunctival congestion arose in dogs with anti-PEG IgM after the injection of sLip (Fig. 7D). There was obvious hemolysis in the centrifugated blood samples of the stimulated group after injection (Fig. 7E). The peripheral blood cell analyses of the blood samples collected at 2 h after injection showed that the hemoglobin content (HGB) and hematocrit value (HCT) in the stimulated dogs were abnormally increased, which may be related to dehydration caused by vomiting and diarrhea (Fig. 7F and G). In addition, it was worth noting that the proportion of basophils (BASO%) in the blood of the stimulated group significantly raised, suggesting allergic responses (Fig. 7H).

The amplified ABC phenomenon and the unexpected adverse reactions by virtue of the existence of anti-PEG IgM were not found in small animals with weak complement capacity but in beagle dogs. Moreover, these adverse actions are quite similar to the infusion reactions reported in the clinical applications of PEGylated liposomes, which suggested that canines may serve as a sensitive clinically relevant model for evaluating the biosafety of PEGylated nanomedicines^44^, ^45^, ^46^.

### 3.6 Clinical relevance: more complex conditions of organisms and distinguishable performances of PEGylated liposomes from model animals

To investigate the clinical relevance of the *in vivo* performance of sLip with inter-individual properties of anti-PEG IgM and complement capacity, a clinical trial of 7 patients with breast cancer (female, aged 34–65) treated by PEGylated liposomal doxorubicin (Duomeisu®) was conducted. The detailed information of each patient was listed in Supporting Information Table S2. The anti-PEG IgM titers in each patient were measured. As shown in Supporting Information Fig. S4B, the anti-PEG IgM titers exhibited diversity across patients and were generally lower in contrast to previously measured levels in healthy subjects, potentially due to underlying pathological factors. Patient #1 and #2 possessed a higher level of anti-PEG IgM in their serum, whose titers were respectively 1:172 and 1:190. The complement capacity of each patient was relatively compared through the quantitation of C3*α* in the serum of each patient on Western blot bands (Fig. S4C and S4D).

**Figure 8:**
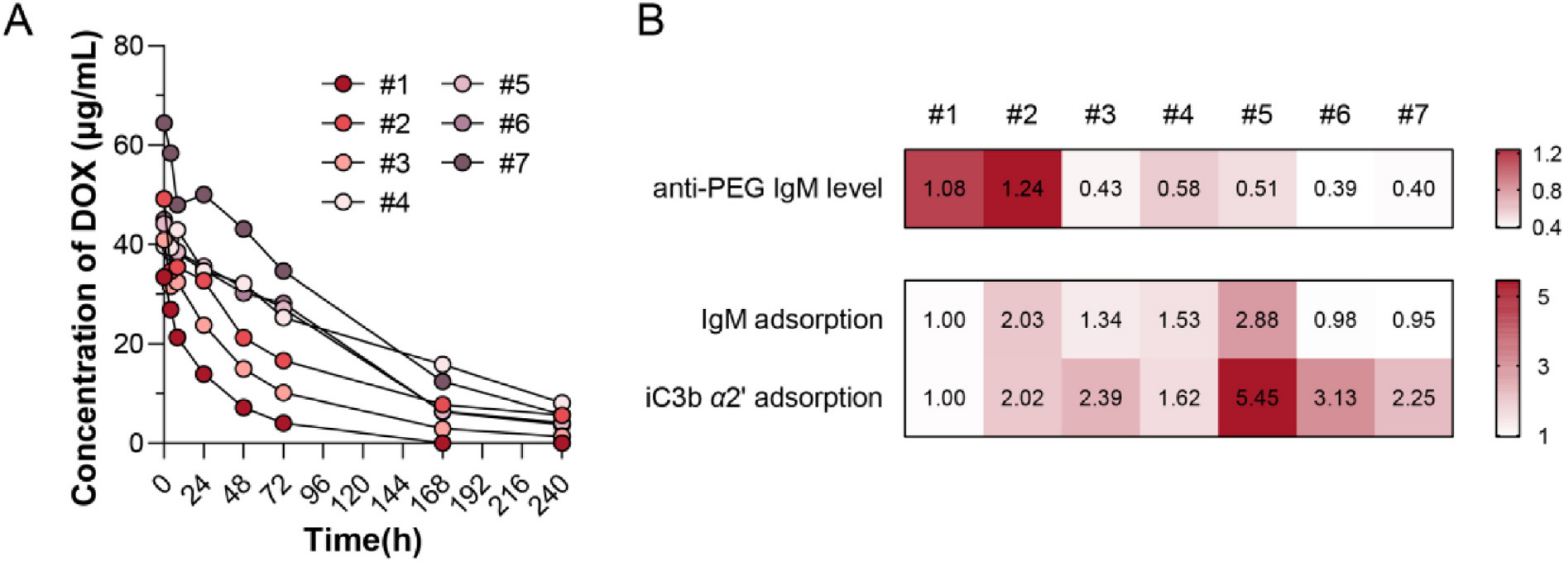
Clinical data of 7 patients with breast cancer administered Duomeisu® (30 mg/m^2^). (A) Plasma concentration of doxorubicin (DOX) after intravenous infusion in each patient. (B) Heat map of anti-PEG IgM level, relative IgM adsorption and relative iC3b *α*2' adsorption among patients. Relative level of Patient #1 was normalized as 1.

In the clinical trial, blood from each patient was sampled at predetermined time points after intravenous infusion of Duomeisu® at a dose of 30 mg doxorubicin/m^2^, which was administered under medical supervision. The plasma contents of Duomeisu® were measured precisely by detecting the total doxorubicin (DOX) concentration using LC‒MS/MS. As displayed in Fig. 8A, the pharmacokinetics profiles of Duomeisu® varied among different patients and the AUC_0–24 h_ ranged from 1076 to 6196 μg·h/mL. The pharmacokinetic parameters of each patient were calculated in Supporting Information Table S4. Meanwhile, the response to the treatment of each patient was recorded, including the therapeutic effect and toxicity, as listed in Table 1.

**Table 1:** Patients’ response to the administration of Duomeisu® (30 mg DOX/m^2^).

Patient ID	Adverse event	Anti-tumor efficacy
#1	Obvious pain (self-report)	PR
#2	Plantar-palmar erythrodysesthesia (Grade I); Oral mucositis (Grade I); Constipation (Grade I)	SD
#3	/	SD
#4	/	SD
#5	Nausea & vomiting	PD
#6	/	PD
#7	/	PD

PR, partial response; SD, stable disease; PD, progressive disease.

To extrapolate the results obtained in animal models to humans, the correlation between the *in vivo* performance of Duomeisu® in clinical practice and the level of anti-PEG IgM and complement was analyzed. It was observed that the rapid clearance of PEGylated liposomes was not necessarily associated with adverse reactions in humans, which was different from that in preclinical animal models. The adverse events in Patient #1 and #2 may be related to their relatively higher titers of anti-PEG IgM in serum. However, there seems to be no explanation for the untoward effect during the infusion of Patient #5. To further investigate the *in vivo* regulating indicators of Duomeisu® and the reason for the different performances, the protein corona of Duomeisu® in the baseline serum of each patient was prepared and analyzed. Surprisingly, as shown in Fig. S4E, the higher absorption of iC3b *α*2’ (one of the cleavages of C3) and IgM on the surface of liposomes may be responsible for the intense adverse reactions of Patient #5. The possible reason was that the anti-PEG IgM in humans was not produced by sLip stimulation as in animal models (pre-existing without injections of PEGylated therapeutics and was highly related to the widespread utility of PEGs in daily necessities, cosmetics, and food), leading to the different specificity to sLip. Also, the human body is more intricate compared to animal models, and there are multiple physiopathological differences among individuals (*e.g.*, the capacity of the immune system, the concentration of dysopsonins in plasma, the course of disease) that cannot be simply generalized^47^.

To visualize the disparities of various indicators among patients, the anti-PEG IgM level, relative IgM adsorption, and relative iC3b *α*2’ adsorption of each patient were shown in the heatmap. The adsorption in the serum of Patient #1 was normalized as 1. As displayed in Fig. 8B, besides the higher titer of anti-PEG IgM, significant cleavage of C3 by liposomes may be a potential indicator to anticipate and mitigate the unfavorable administration of liposomal agents in clinical practice.

## 4 Discussion

In the present work, we focused on the interspecies variation of the *in vivo* fate of PEGylated stealth liposomes (sLip) in order to facilitate clinical translation and promote the safe and rational use of liposome-based therapeutics in clinics. We emphasized the role of anti-PEG antibodies and complements in the elimination of sLip and suggested that the differences in complement capacity resulted in the disparity of *in vivo* performances among mice, rats, and beagle dogs (including the PK and biosafety). Large animals (such as canines) with sufficient complement capacity exhibited a pronounced accelerated blood clearance (ABC) phenomenon and adverse reactions caused by the existence of anti-PEG antibodies, which can be used as a clinical reference.

Firstly, to facilitate a cross-species comparison of the *in vivo* performance of sLip, we calculated and determined dosages based on the interspecies dose exchange index^29^. PEGylated liposomal doxorubicin (PLD), a well-established liposomal agent, is administered at a dose of 20–50 mg doxorubicin/m^2^ in humans. Considering body weight and the PLD formula (approximately 20% w/w doxorubicin/HSPC), the standard dosage in humans is approximately 4 mg HSPC/kg (equivalent to 5 μmol/kg, converted from 30 mg doxorubicin/m^2^). After calculations, the equivalent doses are approximately 50 mg HSPC/kg (66 μmol/kg) in mice, 25 mg HSPC/kg (33 μmol/kg) in rats, and 10 mg HSPC/kg (13 μmol/kg) in beagle dogs. In our study, we focused on the relationship between nanocarrier quantity and the complement capacity. Even for liposomes with varying loads, the carrier count remains consistent due to the same formulation, provided particle size is controlled.

Our results demonstrated that IgM and complement system could synergistically eliminate sLip, but the complement capacity is the bottleneck for the accelerated clearance. Universally acknowledged, IgM and IgG are crucial immunoglobulin isotypes in bodies, so both of them were detected at first. IgM, being the primary antibody secreted during the humoral immune response, is rapidly produced upon infection but has a relatively short duration of action. On the other hand, IgG is the predominant antibody generated by the secondary immune response and exhibits a longer-lasting effect^48^^,^^49^. With its 10 Fab segments and 5 Fc segments, IgM possesses a robust antigen-binding capacity and facilitates easier complement activation compared to IgG^50^. After a week of the stimulation in mice (5 mg lipid/kg), the titer of anti-PEG IgM and anti-PEG IgG in mice was 1:655 and 1:46 relatively (Fig. 1A and Supporting Information Fig. S1), which indicated that the subsequent impact of IgM on the fate of sLip was more significant than that of IgG, at least in this classical modeling approach. Besides the anti-PEG IgM, we highlighted the decisive role of the complement system in the elimination of sLip as a downstream checkpoint. It demonstrated that the insufficiency of complement, even in the presence of sufficient antibodies, led to the IgM-nondependent clearance rate. The ABC phenomenon was significantly weakened when the complement was completely inhibited. All these results showed that complement capacity is the bottleneck in the whole process of accelerated clearance of sLip.

Interspecies differences in complement have been mentioned in previous studies. It was reported that the C3 concentration was approximately 30–40 mg/dL in mice serum^51^^,^^52^, 70 mg/dL in rats^53^^,^^54^ and 100–150 mg/dL in humans^52^^,^^55^. For the first time, we analyzed the relationship between the *in vivo* fate of sLip and interspecies physiological differences and revealed that the differences in complement capacity were responsible for the various interspecies performances of sLip. Due to the limited capacity of the complements in mice, the elimination of sLip was restricted, even in the presence of abundant anti-PEG IgM. However, most of the preclinical studies of liposomes and other nanomedicines were conducted in mice. The ignorance of the defective complement system in small animals may bring inaccuracies and erroneous cognition to the experiment results, which is unfavorable to guide the clinical translation of nanomedicines.

In some previous work, canines were reported to have a high response to PEGylated liposomes and polymeric nanoparticles^28^^,^^56^. Also, our results exhibited that compared to rodent models, some anti-PEG IgM-mediated adverse reactions were only presented in canine models. Though the degree of the response is more intense than that in humans, a similar trend suggested that dogs may be qualified models for warning of some adverse reactions in the preclinical study of PEGylated nanomedicines. In the clinical practice of PEGylated therapeutics, no evident ABC phenomenon was reported at all though pre-existing anti-PEG antibodies may be present in 24%–97.5% of the general population^18^^,^^48^^,^^49^^,^^57^^,^^58^. Nevertheless, anti-PEG antibodies still pose a potential threat to the safe application of PEGylated products for the widespread use of vaccines. Therefore, it is necessary to establish a mature preclinical model and explore the correlation between physiological indicators and *in vivo* behavior.

From the results, we found that sometimes the results of antibody biological activity detected by ELISA and the results of antibody adsorption on the surface of sLip were inconsistent. For example, like Patient #5, the titer of anti-PEG IgM detected by ELISA was not high, but it showed that more IgM was adsorbed in the protein corona formed on the surface of Duomeisu® after incubation with the baseline serum, which caused stronger complement activation. We believed that due to the diverse sources of anti-PEG antibodies in humans, which were different from the designed production on animals. The production of anti-PEG antibodies in healthy humans (without the injection of PEGylated medicines) is mainly related to daily skin exposure, which may respond *via* different immune pathways and choose the particular binding site on PEG in contrast with that caused by the direct injection of animals. Therefore, the detection of IgM adsorbed in the protein corona can more truly reflect the combination of liposomes and IgM, and make a reasonable explanation for the reverse results of ELISA and the characterization of protein corona.

In the present work, we only concentrated on the crucial factors of ABC phenomenon and there were still several interspecies variabilities we have not involved. For instance, the complement activation and C3 regulation mediated by nanomedicines in different species were not the same. Banda et al.^59^ found that nanoworms mainly activate complement through the antibody-mediated alternative pathway, with the lectin pathway assisting activation; while in mouse serum, nanoworms mainly activate complement through the lectin pathway by binding to MBL-A/C. Li et al.^7^ found that dogs and rats tended to activate complement through the classical or lectin pathways, with the alternative pathway assisting, and differences occurred among strains. In general, the regulatory effect of complement on nanomedicines is species-specific and should be given special consideration in preclinical studies. Also, the MPS-related cells are physiologically different cross-species. In mice, rats, dogs, and primates, liver-resident Kupffer cells are the predominant scavenging MPS-related cells, while in pigs, pulmonary intravascular macrophages (PIMs) dominate. This will lead to huge differences in biological distribution and biosafety among species, such as the complement activation-related pseudo allergy (CARPA) caused by nanomedicines reflected as specific pulmonary arterial hypertension in pigs^60^, ^61^, ^62^.

In summary, a better understanding of interspecies variabilities paves the way to predict the efficacy of medicines and prevent adverse reactions through inter-individual differences in some key indicators. The establishment of an appropriate preclinical model for PEGylated nanodrugs is conducive to removing the obstacles to effective evaluation and efficient translation from the lab to the clinic.

## 5 Conclusions

The prevalence of anti-PEG antibodies in humans was thought to be increasingly associated with the widespread use of PEGs and PEG derivatives in various cosmetics, drugs, and food, which may result in ABC phenomenon and other serious adverse effects when PEG-modified therapeutics are administered to patients. However, the fate of PEGylated nanomedicines differed between animal models and humans because of the overlooked physiological variations among species. Therefore, the development and application of PEGylated nanodrugs meet new challenges. In this study, we chose mice, rats, and beagle dogs as animal models to investigate the *in vivo* performance of PEGylated liposomes. We found that the ABC phenomenon existed in all animal species above but the extent varied apparently. The elimination of sLip was also affected by the IgM titers and quantities of nanocarriers. It was verified that anti-PEG IgM and complement systems can synergistically trigger accelerated blood clearance of PEGylated liposomes, and the capacity of the complement system is determined downstream. The differences in concentrations and properties of anti-PEG IgM and complements may lead to the various interspecies performances of liposomes. However, most small animal models (*e.g.* rodent models) lack the human-like complement functionality and can not well simulate the clinical performance of sLip under the circumstance of pre-existing anti-PEG IgM, which may lead to the underprediction of hypersensitivity reactions observed clinically. More predictive preclinical models that accurately reflect the human immune response need to be developed to improve the translation of PEGylated liposomal nanomedicines.

## Author contributions

Ercan Wu and Juan Guan contributed to experiments design and implementation, and manuscript preparation; Yifei Yu, Shiqi Lin, and Zui Zhang contributed to separation methods establishment and sample detection; Tianhao Ding, Yuxiu Chu, Fan Pan, and Yang Yang contributed to pharmacokinetics evaluations and sample collection; Mengyuan Liu contributed to literature research and information collection; Jian Zhang contributed to the collection of clinical data. Jun Qian and Changyou Zhan conceived this research and contributed to the whole work direction and manuscript revision.

## Conflicts of interest

The authors declare no conflicts of interest.

## Appendix A Supporting information

The following is the Supporting data to this article:
