# Supplementary material for: Exemplifying interspecies variation of liposome in vivo fate by the effects of anti-PEG antibodies
Source: Acta Pharm Sin B. 2024 Aug 5;14(11):4994–5007. doi: 10.1016/j.apsb.2024.07.009 (PMC11628802; doi:10.1016/j.apsb.2024.07.009)
Supplement: Multimedia component 1 [file mmc1.pdf]

Supporting Information for

ORIGINAL ARTICLE

Exemplifying interspecies variation of liposome *in vivo* fat e by the effects of anti-PEG antibodies

Ercan Wu[a,b,†], Juan Guan[a,†], Yifei Yu[b], Shiqi Lin[b], Tianhao Ding[b], Yuxiu Chu[b], Feng Pan[a], Mengyuan Liu[a], Yang Yang[b], Zui Zhang[b], Jian Zhang[c,*], Changyou Zhan[b,*], Jun Qian[a,*]

[a]*School of Pharmacy, Key Laboratory of Smart Drug Delivery (Fudan University), Ministry of Education & Department of Pharmacy, Huashan Hospital, Fudan University, Shanghai 201203, China*

[b]*Department of Pharmacy, Shanghai Pudong Hospital, Pudong Medical Center & Department of Pharmacology, School of Basic Medical Sciences & State Key Laboratory of Molecular Engineering of Polymers, Fudan University, Shanghai 200032, China*

[c]*Department of Medical Oncology, Fudan University Shanghai Cancer Center, Shanghai 200032, China*

Received 24 April 2024; received in revised form 18 June 2024; accepted 28 June 2024

[†]These authors made equal contributions to this work.

*Corresponding authors.

E-mail addresses: qianjun@fudan.edu.cn (Jun Qian), cyzhan@fudan.edu.cn (Changyou Zhan), zhangjian1001@csco.ac.cn (Jian Zhang).

Table S1 Characterization of liposomes. Data were means ± SD (*n*=3).

Liposomes	Size (nm)		PDI		Zeta potential (mV)	
	Mean	SD	Mean	SD	Mean	SD
sLip	89.63	3.374	0.042	0.021	−13.7	0.907
sLip/DiD	81.40	7.800	0.180	0.025	−11.5	0.681
Duomeisu®	86.12	1.559	0.120	0.051	−22.6	1.420

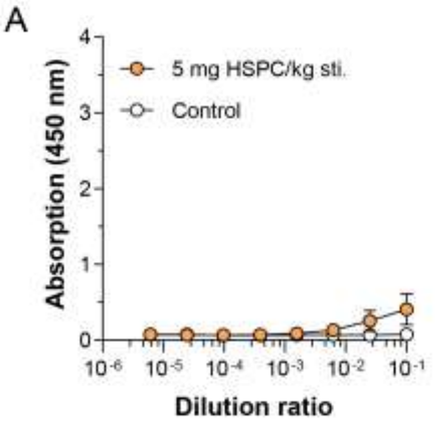

Figure S1 (A) Anti-PEG IgG was stimulated after intravenous administration of sLip (5 mg/kg) and detected 5 days post-injection by ELISA (*n*=12–13). Data were means ± SDs and analyzed by GraphPad Prism 8.0.

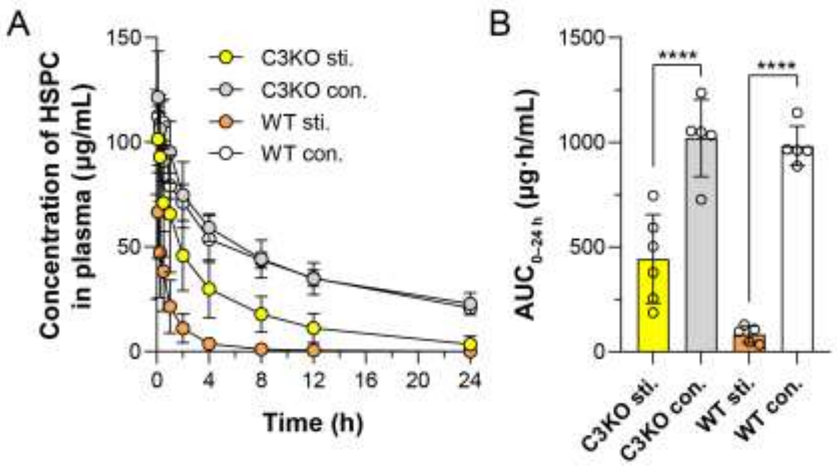

Figure S2 (A) Plasma concentration of sLip at the low dose (5 mg HSPC/kg) in the stimulated mice and control mice (*n*=4–6). (B) The $AUC_{0–24h}$ of sLip was calculated and compared between groups. At the low dose, stimulation with sLip resulted in a 56.5% decrease in $AUC_{0–24h}$ of C3KO mice (443 µg·h/mL *versus* 1019 µg·h/mL) and a 91.6% decrease in that of wide-type mice (81.82 µg·h/mL *versus* 979.5 µg·h/mL) compared to their respective control groups (*n*=5–6). Data are means ± SDs and analyzed by GraphPad Prism 8.0. Statistical significance in the figure (B) was evaluated by t-test (*****P*<0.0001).

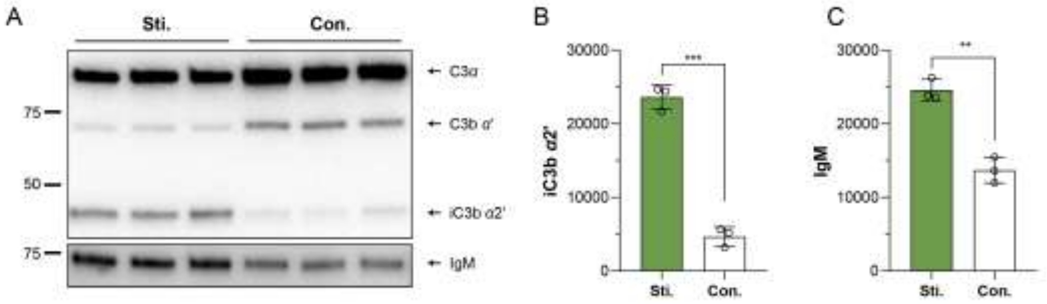

Figure S3 The protein corona of sLip formed in the stimulated and naïve serum of beagle dogs. (A) Western blot analyses of C3α, C3b α', iC3b α2' and IgM in the corona formed on the liposomal surface after incubation with stimulated (Sti.) and naïve (Con.) dog serum for 1 h at 37 °C (n=3). (B) The quantitation of iC3b α2' in the protein corona of sLip formed in dog serum on Western blot bands analyzed by Image J software (n=3). (C) The quantitation of IgM in the protein corona of sLip formed in dog serum (n=3). Data were means ± SDs and analyzed by GraphPad Prism 8.0. Statistical significance in the figure (B–C) was evaluated by t-test (0.001<***P*<0.01, 0.0001<****P*<0.001).

Table S2 Supplemental information of patients[a].

Patient ID	Age	Diagnosis
#1	34	Right breast cancer with metastasis to left breast, chest wall, bones, and lymph nodes (cT4N3M1, stage IV);<br>Type 2 diabetes
#2	65	Left breast cancer after surgery (pT2N3M0, stage III C), with metastasis to liver, pleura, and lymph nodes (rTxNxM1, stage IV);<br>Hypertension;<br>Type 2 diabetes
#3	65	Chest wall cancer recurrence after left breast cancer surgery and right breast cancer surgery, with metastasis to liver, lung, pleura, bones, and lymph nodes (rTxNxM1, stage IV);<br>Chronic hepatitis B;<br>Possible lymphangitis carcinomatosa
#4	38	Chest wall recurrence after left breast cancer after surgery (pT2N3M0, stage III C), with metastasis to lymph nodes (rTxNxM1, stage IV)
#5	54	Left breast cancer after surgery, with metastasis to lung and lymph nodes
#6	51	Left breast cancer after surgery (pTxN0M0), with metastasis to liver, lung, bones, and lymph nodes (rTxN3M1, stage IV);<br>Hypertension

#7	56	Right breast cancer after surgery, with metastasis to liver, lung, pleura, bones, and lymph nodes (rTxN3cM1, stage IV);<br>Malignant pleural effusion

[a]All patients received their first injection of PEGylated liposomal medication.

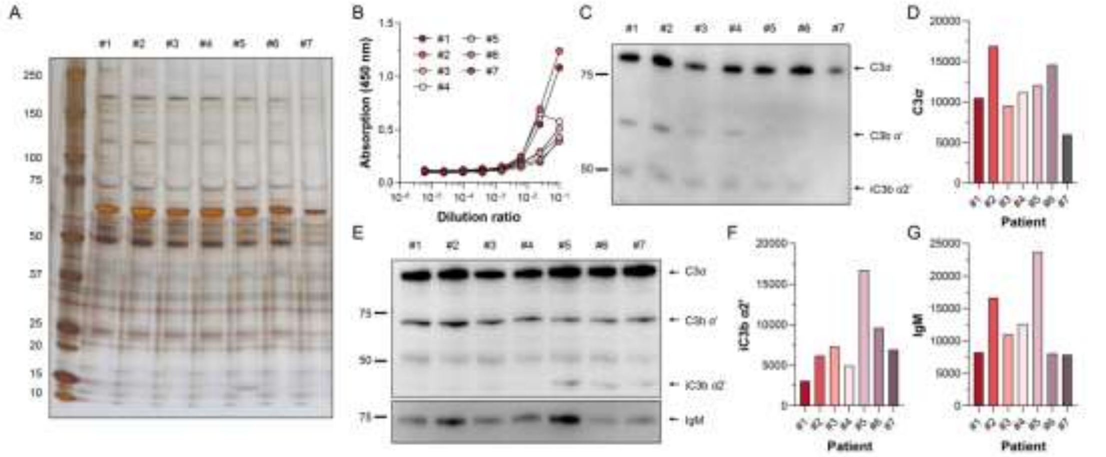

Figure S4 The level of IgM and complement system in 7 patients. (A) Proteins in the baseline serum of each patient (1,000 times diluted) was separated by SDS-PAGE and compared by Fast Silver Stain Kit. (B) The anti-PEG IgM level in the baseline serum of each patient was measured by ELISA. (C) Western blot analyses of C3α, C3b α’, iC3b α2’ in the baseline serum of each patient (1,000 times diluted). (D) The quantitation of C3α in the serum of each patient on Western blot bands analyzed by Image J software. (E) Western blot analyses of C3α, C3b α’, iC3b α2’ and IgM in the corona formed on the surface of Duomeisu® after incubation with the baseline serum of each patient for 1 h at 37 °C. (F) The quantitation of iC3b α2’ in the protein corona of Duomeisu® formed in the serum of each patient. (G) The quantitation of IgM in the protein corona of Duomeisu® formed in the serum of each patient.

Table S3 Supplemental pharmacokinetic parameters of sLip/DiD in animals[a].

Animal		Dose (mg HSPC/kg)		Model	Pharmacokinetic parameters			*n*
Species	Strain	Stimulation	Administration		$t_{1/2\alpha}$ (h)	$t_{1/2\beta}$ (h)	Cl (mL/h)	
Mouse	ICR	/	50	CA, IV bolus, 2 compartments	1.814±0.541	20.604±2.679	0.062±0.007	4
		0.5			1.643±0.994	14.848±1.938	0.065±0.027	4
		5			1.740±0.285	18.388±2.86	0.072±0.012	4
		50			1.357±0.899	25.788±12.647	0.053±0.016	4
Rat	SD	/	25		2.991±1.163	26.260±8.092	0.417±0.091	4
		0.25			0.016±0.005	3.369±1.557	4.015±3.123	3
		2.5			2.323±2.669	6.127±2.920	1.414±0.476	4
		25			1.076±1.034	16.581±7.690	0.431±0.274	4
Mouse	ICR	/	5		1.733±0.554	26.550±8.09	0.054±0.014	4
		0.5			0.928±0.632	7.787±6.470	0.396±0.321	4
		5			0.961±0.565	11.358±6.165	0.202±0.110	4
		50			1.336±0.516	21.977±11.328	0.090±0.037	4
Rat	SD	/	2.5		3.400±4.987	17.013±1.161	0.702±0.057	4
		0.25			Not suitable			4
		2.5						4
		25			0.660±0.431	18.783±28.500	5.714±2.816	4
Mouse	C57BL/6J -C3KO	/	50		1.544±1.031	20.265±5.139	0.049±0.006	5
		5			1.600±0.955	25.438±13.975	0.054±0.019	4
		/	5		1.605±0.499	19.325±4.365	0.063±0.012	5
		5			0.850±0.614	6.378±2.798	0.272±0.158	6
Canine	Beagle	/	10		11.078±17.314	32.433±4.808	11.609±1.169	4
		1			0.213±0.110	30.970±5.556	390.223±53.194	4

[a]All parameters were calculated by PK Solver 2.0. Fitting weight W= 1/C^2_pre.

Table S4 Supplemental pharmacokinetic parameters of Duomeisu® in patients[a].

Patient ID	Dose (mg DOX/m^2)	Infusion time (h)	Model	Pharmacokinetic parameters		
				$t_{1/2\alpha}$ (h)	$t_{1/2\beta}$ (h)	Cl (mL/h)
#1	30	7.217	CA, IV infusion, 2 compartments	2.400×10^{-5}	44.906	45.882
#2		3.667		0.049	85.858	12.475
#3		1.450		0.391	50.981	16.636
#4		1.167		0.133	105.781	8.541
#5		3		0.055	68.533	12.781
#6		1.117		0.129	64.847	10.659
#7		1		0.138	71.482	9.170

[a]All parameters were calculated by PK Solver 2.0. Fitting weight W= 1/C^2_pre.
